# Sendai virus-based liposomes enable targeted cytosolic delivery of nanoparticles in brain tumor-derived cells

**DOI:** 10.1186/1477-3155-10-9

**Published:** 2012-02-17

**Authors:** Veronica Dudu, Veronica Rotari, Maribel Vazquez

**Affiliations:** 1 The City College of New York, Department of Biomedical Engineering, 160 Convent Avenue, New York, NY 10031, USA

**Keywords:** Virus-based liposomes, Quantum dots, cancer, EGFR, Sendai Virus

## Abstract

**Background:**

Nanotechnology-based bioassays that detect the presence and/or absence of a combination of cell markers are increasingly used to identify stem or progenitor cells, assess cell heterogeneity, and evaluate tumor malignancy and/or chemoresistance. Delivery methods that enable nanoparticles to rapidly detect emerging, intracellular markers within cell clusters of biopsies will greatly aid in tumor characterization, analysis of functional state and development of treatment regimens.

**Results:**

Experiments utilized the Sendai virus to achieve *in vitro*, cytosolic delivery of Quantum dots in cells cultured from Human brain tumors. Using fluorescence microscopy and Transmission Electron Microscopy, *in vitro *experiments illustrated that these virus-based liposomes decreased the amount of non-specifically endocytosed nanoparticles by 50% in the Human glioblastoma and medulloblastoma samples studied. Significantly, virus-based liposome delivery also facilitated targeted binding of Quantum dots to cytosolic Epidermal Growth Factor Receptor within cultured cells, focal to the early detection and characterization of malignant brain tumors.

**Conclusions:**

These findings are the first to utilize the Sendai virus to achieve cytosolic, targeted intracellular binding of Qdots within Human brain tumor cells. The results are significant to the continued applicability of nanoparticles used for the molecular labeling of cancer cells to determine tumor heterogeneity, grade, and chemotherapeutic resistivity.

## Background

Nanoparticles have facilitated unprecedented study of biological processes and molecular markers within a variety of cell samples (reviewed in [1-4]). Diagnostic assays where nanoparticles are used to detect the presence and/or absence of a combination of cell markers are becoming increasingly significant in the identification of progenitor or stem-like cells found within a variety of tumors [5]. While nanotechnology has pioneered major advances in cancer detection, diagnosis, and treatment [6], tumors within brain continue to pose one of the lowest survival rates five years after diagnosis [7]. While such poor prognosis is largely associated with the highly invasive nature of malignant brain tumors [8-10], the cellular heterogeneity of diseased brain also plays a large role, as constituent subpopulations of neoplastic cells with stem-like properties [11] appear to be resistant to conventional radiotherapy and chemotherapeutic regimens [12]. Emerging studies have underscored the significance of intracellular markers when identifying neoplastic stem-like populations (reviewed in [13]), either in tandem with existing extracellular markers (e.g. CD133, PAX6, reviewed in [14]) or alone. Numerous cytosolic molecules currently serve as therapeutic targets for radiosensitization, including heat shock proteins [15], binding proteins [16], Hypoxia Inducible Factors HIF1 and HIF2 [17], transcription factors [18], and phospholipoases [19]. In addition, recent studies point to cytosolic markers as excellent detectors of biochemical signatures from cells previously thought to evade the neural system, such as the prion-like protein Doppel (Dpl) found in the male reproductive system [20], and light neurofilament proteins and class III β-tubulin found in bone marrow-derived mesenchymanl stem cells [21].

Labeling of intracellular molecules is notoriously difficult to achieve using nanoparticles because of the highly esoteric selectivity required [22]. Intracellular delivery of nanoparticles is strongly affected by both the nature of the particle and the type of cell examined (reviewed in [23]). For example, established delivery methods of bioconjugates, such as Quantum dots (Qdots), via endocytosis, pinocytosis and injection are known to alter cell function as well as exhibit varied effectiveness per cell type and/or experimental condition [24,25]. Further, alternative approaches such as electroporation [26], nanoneedles [27], and cell-penetrating peptides [28] have led to internalized Qdots that can become trapped within the endocytic pathway and/or form large aggregates in the cytoplasm [29]. Most recently, researchers have utilized cell penetrating peptides [30,31], pH-dependent fusogenic peptides [32], as well as logic-embedded vectors [33] to achieve endosomal release after internalization. Others have minimized endosomal trapping by using silica [34], gold [35,36], and polymer-based nanoparticles [37] and polyactic acid [38], while yet others have disrupted endocytosis by using light-activated disruption of intracellular vesicles [39], or controlled sub-cellular damage of endosomal structures [40].

Recent applications have revived the practice of nanoparticle encapsulation by incorporating nanoparticles within patented synthetic proteins and polymers, as well as within antiretroviral complexes [41], each with a varying degree of endosomal escape. Our group has previously shown that cationic liposomes are able to facilitate intracellular delivery of Qdots within live brain cancer cells [42], but demonstrated that the method is cell line-dependent: Liposomal delivery of Qdots was cytoplasmic within glioblastoma-derived cells, but resulted in endocytosis and trapping of liposomes within endosomes when HeLa cells were used. More unconventional approaches to nanoparticle delivery have begun to incorporate viruses previously used to deliver other nanosized molecules, such as DNA, synthetic oligonucleotides, and pharmaceuticals [43]. Chymeric bacteriophages have been employed to target tumors and introduce intracellular agents bound to its surface [44], while the plant mosaic virus [45] was used to incorporate Qdots coated with various molecules (e.g. streptavidin-biotin, dihydrolipoic acid) within its capsid. A recent study adapted the simian virus 40 capsid to encapsulate Qdots functionalized with different surface coatings (e.g. DNA, PEG) for transport within kidney cells [46]. While delivery was successful, it remained unclear whether the virus itself enabled cytosolic release of Qdots or if the Qdots remained trapped within cellular compartments [46].

The current study has achieved cytoplasmic delivery of targeted Qdots via chimeric fusions between the Sendai virus and cationic liposomes [47]. The Sendai virus is a mouse parainfluenza virus that has been safely used for over two decades, *in vitro *and *in vivo*, to deliver molecules such as plasmid DNAs, siRNAs, proteins, iron particles, and pharmaceuticals into numerous cell types (reviewed in [48]). Its role as a delivery vector capitalizes on two types of proteins present in its capsid: (i) Hemagglutinating and Neuraminidase (HN) proteins, used for attachment of the virus to neuraminic acid-containing receptors on host cells, hemagglutination of erythrocytes, and neuraminidase activity [49]; and (ii) Fusion (F) proteins needed for virus penetration of host cell membranes, virus-induced hemolysis, and cell fusion [49-51]. In this work we use the Sendai virus to generate virus-based liposomes that achieve cytosolic delivery of targeted Qdots into live Human brain tumor cells with high, consistent efficiency. Qdots were functionalized with a monoclonal biotinylated antibody (Ab) designed to specifically recognize an intracellular epitope of the Epidermal Growth Factor Receptor (EGFR). EGFR was chosen as a candidate target protein because its over-expression and up-regulation is recognized as a significant step in the pathogenesis and progression of a wide variety of cancers, including tumors of the brain [52-55]. Further, previous work from our laboratory has successfully labeled activated EGFR populations in live brain cancer cells by binding Qdots to the extracellular domain of EGFR and then inducing receptor activation to detect intracellular, activated EGFR [56]. In the current study, delivery of Qdot by chimeric fusions between the Sendai virus and cationic liposomes, henceforth called virus-based liposomes (VBLs), was assessed using three different Human cancer cell types: (i) Medulloblastoma (MB), the most common form of pediatric brain tumor; (ii) Glioblastoma (GBM), the most common form of tumor in adult brain; and (iii) HeLa cervical cancer, a well-studied cell line used here as an experimental control.

## Results and Discussion

### Cellular EGFR Localization in Medulloblastoma

In order to examine the targeted delivery of nanoparticles to cytosolic EGFR targets in MB, experiments first examined EGFR in MB cells during signaling events, with and without ligand stimulation. EGFR signaling is known to be tightly regulated by receptor endocytosis and lysosome-mediated degradation [57]. In adult brain, the tumor suppressor gene, Mig-6, has been recently shown to quell the malignant potential of GBM and dampen EGFR signaling by driving EGFR into late endosomes and lysosome-mediated degradation upon ligand stimulation [58]. In pediatric brain, EGFR signaling within more embryonal MB cells delineates a poorer outcome, and is believed to be a function of MB type and grade [52]. Early detection of such signaling is highly significant for MB patient prognosis, as 70% of children with elevated levels of EGFR expression succumb to their neoplastic disorder prior to 4 years of age [52].

**Figure 1:**
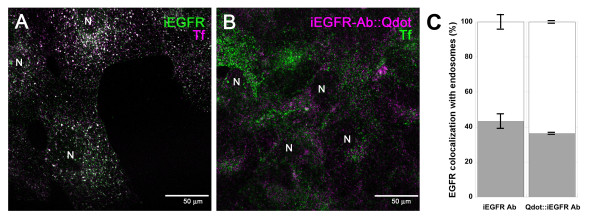
**Immunostaining of intracellular EGFR in medulloblastoma**. **A**. Labeling of EGFR using an antibody that recognizes an internal isotope, iEFGR (Alexa 488; green), and of the endocytic pathway using Transferrin (Tf) (purple) in medulloblastoma (MB) cells; **B**. Labeling of EGFR in MB using Qdots functionalized with biotinylated iEFGR (purple) and of the endocytic pathway using Tf (green); **C**. Percentage co-localization between Tf and iEGFR Ab, and Tf and Qdot:iEGFR Ab complexes. Nuclei are indicated by the letter "N". Scale bar equals 50 μm in both confocal images.

**Figure 2:**
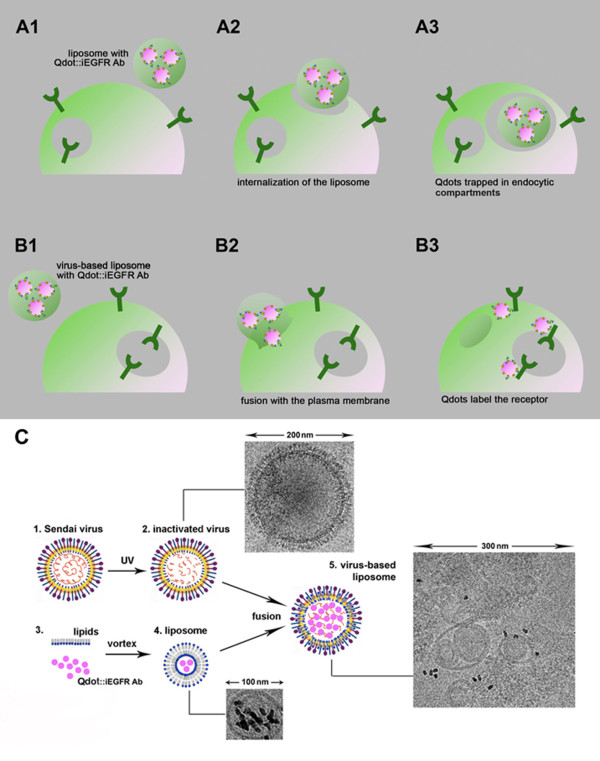
**Schematics of nanoparticle delivery using liposomes-only and virus-based liposomes**. **A**. Intracellular delivery of Qdots functionalized with biotinylated antibodies for iEFGR by using liposomes-only. Liposomes containing functionalized Qdots are incubated with the cells (A1). The liposomes are internalized via endocytosis by the cells (A2). Liposomes are internalized within cell endosomes, trapping the functionalized Qdots (A3); **B**. Intracellular delivery of Qdots functionalized with biotinylated antibodies for iEFGR by using virus-based liposomes (VBLs). VBLs containing functionalized Qdots are incubated with the cells (B1). VBLs fuse with the plasma membrane to release Qdots into the cytosol (B2). The functionalized Qdots bind to their intracellular targets on the EGFR (B3). **C**. Preparation of VBLs: Sendai viruses (1) were inactivated by exposure to UV light (2) (for details see Methods). A TEM insert illustrates an inactivated virus of ~ 200 nm diameter. Liposomes containing targeted Qdots (4) were prepared as described in Methods by incorporating Qdots functionalized with anti-iEGFR antibodies into lipidic membranes (3). A TEM insert illustrates a ~100 nm diameter liposome containing functionalized Qdots. Next, the liposomes were fused with the inactivated viruses, to give rise to VBLs (5). A TEM insert illustrates the fusion products.

Our first set of experiments examined EGFR localization within MB-derived cells by labeling the receptor with an antibody that recognizes the intracellular amino-acid sequence 985-996 of EFGR, henceforth referred to as iEGFR. This domain was chosen for experiments of targeted delivery so that Qdot binding would have minimal effects on EGFR phosphorylation and its subsequent recruitment of signaling adaptor proteins (see Methods). EGFR location was determined both by standard immunostaining using secondary fluorescence Alexa 488 detection (Figure 1A), as well as via nanoparticle complexes formed between biotinylated iEFGR Abs and Qdots (Figure 2B). Measured co-localizations of the receptor with the endocytic pathway via confocal microscopy (Figure 1A) illustrated that 43.1% +/- 4.4% of EGFR was present within the endosomes of non-ligand stimulated MB cells when measured via conventional secondary antibodies, consistent with 36.2% +/- 0.4% endosomal presence when measured via Qdots (Figure 1C).

These initial results are not only among the first to utilize Qdots to measure the cytosolic EGFR population in MB, but additionally depict the EGFR population internalized within the endocytic compartments of these cells in the absence of tyrosine kinase activity [59]. These findings become critical to nanoparticle labeling of EGFR within MB, as increased endosomal EGFR signaling is being explored as a marker of de-differentiated cells that are linked to recurrence and radioresistivity in malignant brain tumors [52].

### Nanoparticle Cytosolic Delivery via Liposome-only and Virus-Based Liposomes

**Figure 3:**
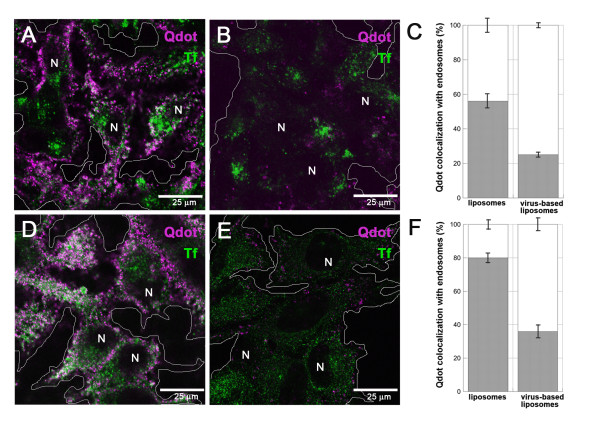
**Intracellular delivery of Qdots using liposomes-only and virus-based liposomes in glioblastoma and HeLa cells**. Cell nuclei are denoted by the letter "N" and endocytic pathways are labeled by Transferrin (Tf) (green). Scale bar equals 25 μm in all confocal images. **A**. Double labeling of glioblastoma (GBM) cells for non-targeted Qdots delivered by using liposomes-only (purple). **B**. Double labeling of GBM cells for non-targeted Qdots delivered by using virus-based liposomes (VBLs) (purple). **C**. Measurement of Qdot co-localization with endosomes (black) and cell cytosol (white) when delivered by liposomes-only or VBLs in GBM; **D**. Double labeling of HeLa cells for non-targeted Qdots delivered by using liposomes-only (purple). **E**. Double labeling of HeLa cells for non-targeted Qdots delivered by using VBLs (purple). **F**. Measurement of Qdot co-localization with endosomes (black) and cell cytosol (white) when delivered by using liposomes-only and VBLs.

Researchers have recently illustrated that nanoparticles conjugated to a drug or antibody cannot simply be internalized in order to bind cytosolic or endosomal targets, because the nanocomplexes can distinctly alter cellular processes at the molecular level [60]. As a result, the next set of experiments utilized VBLs to facilitate Qdot binding to cytosolic iEGFR in order to derive measurements of both the populations of native cytosolic EGFR and endosomal EGFR within MB samples. Here, we examined the *in vitro*, intracellular delivery of Qdots targeted to iEGFR via cationic liposomes alone, and by using VBLs. (Note that experiments do not seek to track the translocation of cytosolic EGFR proteins to endosomes during signaling). As shown in the schematics of Figure 2, when liposomes are used for intracellular delivery (Figure 2A1), Qdots can become internalized non-specifically within endocytic compartments (Figure 2A2) and remain trapped within liposomes, which are themselves trapped within the endocytic compartments of the cell (Figure 2A3). By contrast, when VBLs are used for intracellular delivery (Figure 2B1), functionalized Qdots can be released within the cytosol (Figure 2B2) and remain free to bind to their targets (Figure 2B3). A schematic of how the VBLs were generated (Figure 2C) helps to illustrate how VBLs can be selective for intracellular targeting. The cartoon includes TEM images of an inactivated virus (approximately 200 nm in diameter) as well as of a liposome (approximately 100 nm in diameter) that contains Qdots within its lipid membrane. Experiments used both liposomes-only and VBLs to deliver non-functionalized Qdots (i.e. without antibody conjugation) within GBM and HeLa cell samples. Note that experiments were used to measure the differences in cytosolic delivery when using VBLs versus liposomes alone within each cell type, individually. The data is not compared across different cell lines, which invariably have different levels of native EGFR expression. In both samples, the majority of the liposomes containing Qdots were trapped within endosomes as measured previously by our laboratory [42,56] using Qdot-Tf co-localization via confocal microscopy: 56% +/- 4.8% for GBM (Figures 3A and 3C), and 80% +/- 3.5% for HeLa (Figures 3D and 3F). Conversely, when VBLs were used, Qdots were distributed sparsely within the cytosol of GBM (Figure 3B) and HeLa cells (Figure 3E). Measurement of Qdot co-localization with Tf-labeled endosomes confirmed that 75% +/- 1.9% of the Qdots were detected intracellularly and outside of the endosomes for GBM (Figure 3C), leaving only 25% +/- 1.9% trapped in the endosomes. Similarly, 64% +/- 4% of Qdots were detected outside of the endosomes of HeLa cells (Figure 3F), while 36% +/- 4% of Qdots were observed within the endosomes of these cells. Additional co-localization statistics were performed using Manders coefficients with values of 0.48 for GBM and 0.81 for HeLa when liposomes-alone where used with Tf-labeled endosomes. When VBLs were used with Tf-labeled endosomes, Manders coefficient values of 0.72 for GBM and 0.59 for HeLa were tabulated. We note that while the concentration of Qdots delivered using liposomes-only appears higher than the concentration of Qdots delivered via VBLs, we believe this is due to the higher background noise of clustered Qdots endocytosed via the liposomes-only delivery.

Previous studies have utilized nanoparticles for the detection of markers within breast, prostate and lung [61,62], but few have examined Qdot applications in malignant brain tumors [63]. The results of the present study document an increase of up to 50% in the number of intracellularly delivered Qdots that remain free to bind cytosolic targets within malignant brain tumors when compared to liposomes alone. Such dramatic increases will augment detection in numerous cancer diagnostic assays used to characterize the type, grade, and level of heterogeneity in tumors.

### Cytosolic Virus-based Liposome Delivery of Nanoparticles Targeted to iEGFR

**Figure 4:**
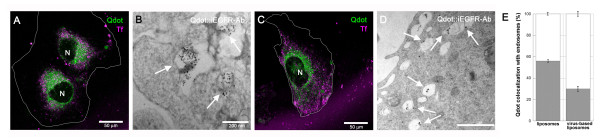
**Delivery of Qdots, targeted and non-targeted to intracellular EGFR, by using liposomes-only and virus-based liposomes in medulloblastoma**. Nuclei are denoted by the letter "N" and endocytic pathways are labeled by Transferrin (Tf) (green) in all confocal images. **A**. Double labeling of medulloblastoma (MB) cells for non-targeted Qdots (purple) delivered by liposomes-only. Scale bar equals 50 μm; **B**. TEM of MB cells treated with liposomes-only, which encapsulated Qdots functionalized with biotinylated antibodies for iEFGR (Qdot::iEFGR Ab complexes). Arrows point to clustered Qdots co-localized with endosomes. Scale bar equals 200 nm; **C**. Double labeling of MB cells for non-targeted Qdots (purple) delivered by using virus-based liposomes (VBLs.) Scale bar equals 50 μm; **D**. TEM of MB cells treated with VBLs that encapsulated functionalized Qdot::iEFGR Ab complexes. Arrows point to dispersed Qdots. Scale bar equals 200 nm. **E**. Measurement of Qdot co-localization with endosomes (black) and with cell cytosol (white) when delivered by liposomes-only or VBLs.

The final set of experiments utilized VBLs to deliver nanoparticles within live MB samples and to image the cellular location of the Qdot::iEGFR Ab complexes via confocal microscopy and/or TEM (Figure 4). When using liposomes-only as the delivery system, non-functionalized Qdots were detected within Tf-labeled endosomes (Figures 4A). Measurement of co-localized fluorescent signatures revealed that 56% +/- 1.4% of Qdots were present within the cell endocytic pathway (Figure 4E). This data indicates that liposomes were predominantly internalized via endocytosis in MB, as has been reported for other cell lines (reviewed in [64]). Moreover, TEM of MB samples treated with liposomes containing Qdot::iEGFR Ab complexes led to detection of the well-known electron-dense cores of the Qdots within the cell endocytic compartments (Figure 4B). In contrast, when Qdots were delivered using VBLs, confocal microscopy illustrated Qdots that were evenly distributed throughout the MB cytosol (Figures 4C). Measured localization of these Qdots revealed that only 30% +/- 3.6% of the Qdot signal was present within the cell endosomes (Figure 4E). Note that Qdots located within the endosome are unlikely to bind iEGFR because they remain trapped within liposomal membranes, as previously shown [42]. However, the data do illustrate that VBLs released the majority of their cargo (i.e. 70% +/- 3.7%) into the cytosol of MB cells. Consistent Manders coefficients of 0.68 support this co-localization. In addition, TEM of the samples treated with VBLs containing functionalized Qdot::iEFGR Ab complexes illustrated that the Qdots indeed labeled iEGFR throughout the cells post VBL delivery (Figure 4D). These novel findings are the first to successfully utilize VBLs for intracellular, nanoparticle delivery, and also the first to achieve cytosolic binding of intracellular targets significant to brain cancer research and detection.

## Conclusions

Intracellular delivery of functionalized nanoparticles is a critical goal for numerous biomedical applications, especially the early detection and diagnostic of malignant brain tumors. Delivery methods that enable Qdots to target specific intracellular brain cancer markers will greatly aid in the molecular labeling of tumor samples [65,66], as well as in surgical imaging procedures of brain tumors [67,68]. The current study is the first to use Sendai virus-based carriers for cytosolic delivery of targeted nanoparticles, as well as the first to explore VBLs for the study of intracellular markers in malignant brain tumors. Results illustrate that VBLs increased the specific intracellular labeling of EGFR by 50%, and, importantly, significantly bypassed Qdot entrapment within endosomes for the GBM and MB brain tumor samples studied. VBLs provide a reliable and consistent method for cytosolic delivery of nanoparticles that are targeted towards highly selective intracellular protein sequences. Further, virus-based liposomes do not only facilitate cytosolic delivery, but additionally provide the feasibility of TEM to enable precise localization of target proteins.

## Methods

### Cell Culture

The medulloblastoma-derived Daoy cell line (#HTB-186, purchased from ATCC, Manassas, VA) was established from a tumor biopsy of a 4-year-old boy. The glioblastoma-derived U251 cells were a kind gift from Dr. Eric Holland (MSKCC) [55]. The cervical cancer-derived HeLa cell line (# CCL-2™) was purchased from ATCC, Manassas, VA. Daoy cells were cultured with sterile EMEM (Mediatech Inc., Herndon, VA), supplemented with 2% L-Glutamine (Mediatech Inc., Herndon, VA), 1% Penicillin-Streptomycin-Amphotericin B - 100× solution (Mediatech Inc., Herndon, VA), and 10% fetal bovine serum (Gemini Bio-Products, West Sacramento, CA). U251 and HeLa cells were cultured with sterile DMEM (Sigma, St. Louis, MO), supplemented with 1% Penicillin-Streptomycin solution (Mediatech Inc., Herndon, VA), and 10% fetal bovine serum (Thermo Scientific HyClone, Logan, UT). The cells were grown onto sterile polystyrene tissue culture flasks (BD Biosciences, Franklin Lakes, NJ) and incubated at 37°C with 5% CO_2_.

### Antibodies and Immunocytochemistry

HeLa, medulloblastoma- and glioblastoma-derived cells grown on coverslips were fixed with paraformaldehyde (Sigma, St. Louis, MO) and labeled with biotinylated mouse antibody for iEFGR (1:500) - recognizing an internal epitope (Biodesign, Saco, ME), and goat anti-mouse AlexaFluor^® ^488 (Invitrogen Molecular Probes, Eugene, OR). For the Qdot labeling, a solution of 5 nM streptavidin-conjugated Qdot 655 (Invitrogen Molecular Probes, Eugene, OR)::5 nM iEGFR antibody was incubated on a shaker for 1 hour at 25°C, and used in the immunocytochemistry after fixing. Transferrin AlexaFluor^® ^488 or 594 conjugates (Tf) (Invitrogen Molecular Probes, Eugene, OR) were used to label the clathrin-mediated pathway: a 20 μg/mL solution of Tf was applied and incubated with the cells for 1 hour at 37°C. After labeling, the cells were subsequently mounted in glycerol (Polyscience Inc., Warrington, PA). Each experiment was performed three times.

### Fluorescence Microscopy and Analysis

Confocal laser scanning microscopy imaging was performed using a Leica TCS SP2 instrument (Leica Microsystems, Bannockburn, IL) with a 63× oil immersion objective (NA 1.4). Identical imaging conditions were used for each set of experiments. A total of 3-5 samples were prepared and three random regions were imaged per sample, three times each per experimental condition. Analysis of confocal images was performed using Matlab software (version 7.7.0.471) to import data as matrices containing absolute intensity values. Data in each matrix was thresholded at 10-15% of the maximum intensity in the respective matrix in order to eliminate background signal due to scatter. Co-localization of fluorescent labels was then defined as the exact overlapping of data points with values above the threshold value of the respective matrices. Thresholded Manders Coefficients were also calculated using NIH Image Software (NIH, Bethesda, MA) to additionally determine co-localization [69].

### Liposome Preparation

Cationic lipids were purchased from Avanti Polar Lipids, Inc. (Alabaster, AL). Two milligrams each of powdered lipids 1-oleoyl-2-palmitoyl-*sn*-glycero-3-phosphocholine (OPPC) and 1, 2 dioleoyl-*sn*-glycero-3-ethylphosphocholine (DOPC+) were dissolved in chloroform at a 1:1 molar ratio in a glass vial. The lipid mixture (1 mg/mL) was aliquoted into glass vials using a Pasteur pipette and the vials were placed in a dessicator connected to a vacuum pump (Neuberger Inc., Trenton, NJ) for 2.5 to 3 hours. Inert nitrogen gas was used afterwards to flush out the dessicator before opening. The vials containing lipid sheets were placed at -20°C for at least 24 hours. The lipid sheets were placed at room temperature prior to use in order to prevent condensation. The lipid sheets were hydrated with either of the following two solutions yielding in both cases liposome solutions with lipid concentration of 0.5 mg/mL: (i) PBS with 5 nM streptavidin-conjugated Qdot 655; (ii) PBS with 5 nM streptavidin-conjugated Qdot 655 functionalized with a biotinylated antibody for iEFGR (Biodesign, Saco, ME). The vials were vortexed for four minutes, and then subjected to 3 cycles of freezing at -20°C and thawing at room temperature. After the last freeze-thaw cycle, the glass vials containing hydrated liposomes were placed at room temperature in order to prevent condensation. The sample was loaded into a 1000 μL gas-tight syringe, which was placed into an Avanti Mini-Extruder (Avanti Polar Lipids, Inc., Alabaster, AL). A polycarbonate membrane with a pore diameter size of 200 nm was used for the extrusion.

### Virus-Based Liposome Preparation

The protocol used in this work was a modified version of the Sendai virus-based protocols found in existing literature [47]. Briefly, commercial, purified Sendai viruses (Charles River, Wilmington, MA) were inactivated by exposure to 9.6 × 105 μJ/cm^2 ^UV light. Inactivated viruses were mixed with liposomes prepared as described above at a ratio of virus: liposomes = 3000 HAU: 1 mg lipid. The mixture was incubated for 10 minutes on ice to allow the viruses to dock onto the liposomes, and afterwards 1 hour at 37°C in a water-bath with shaking (120/min). After this incubation, the Sendai virus-liposome preparation was layered over a sucrose gradient (30% sucrose over 60% sucrose in a 4:1 volume ratio; in PBS), and spun at approximately 22,000 rpm for 3 hours at 4°C. After spinning, the virus-based liposomes were located in a layer between the PBS solution and 30% sucrose, while unincorporated Sendai virus particles were found between the 30% and 60% sucrose layers. The virus-based liposomes were generally used immediately after preparation, or were stored at 4°C for up to 24 hours prior to use.

### Liposome- and Virus-Based Liposome Cell Incubation

The liposomes or virus-based liposome solutions, containing either functionalized or non-targeted Qdots, were applied directly onto cells plated on coverslips. After 10 minutes incubation on ice to allow the docking of the delivery system to the cells, cells were incubated at 37°C for 1 hour. For the intracellular localization of non-targeted Qdots, cells were also incubated with a 20 μg/mL solution of Tf -594 conjugate for 30 minutes at 37°C. After incubation, cells were fixed with 3.7% paraformaldehyde (Sigma, Atlanta, GA) and mounted in glycerol (Polyscience Inc., Warrington, PA).

### Transmission Electron Microscopy (TEM)

For the analysis of intracellular distribution of Qdots, medulloblastoma cells cultured on collagen-coated plates were labeled with Qdot::iEFGR Ab via virus-based liposome delivery, as described above. After washing three times with PBS, the cells were fixed with 2% glutaraldehyde (Electron Microscopy Services, Hatfield, PA) for 2 hours at 4°C and then post-fixed with 1% osmium tetroxide (Electron Microscopy Services, Hatfield, PA) for 2 hours at 4-8°C. After dehydration by immersing in serially diluted aqueous ethanol solutions, the specimens were embedded in epoxy resin, sectioned to 80-100 nm thick, stained with uranyl acetate, and examined with a Zeiss EM 902 (Carl Zeiss, Peabody, MA). As a control, non-targeted Qdots treated cells were also imaged following preparation as described above. A total of 10-15 images per sample were acquired from three different samples per experimental condition.

## List of abbreviations

EGFR: Epidermal Growth Factor Receptor; GBM: Glioblastoma; MB: Medulloblastoma; Qdots: Quantum dots; TEM: Transmitted Electron Microscopy; Tf: Transferrin; VBL: Virus-Based Liposome.

## Competing interests

The authors declare that they have no competing interests.

## Authors' contributions

VD participated in the study design and carried out the EGFR testing of cell samples, immunoassays, nanoparticle delivery and drafted the manuscript. VR carried out quantitative analysis of data images using Matlab and NIH Image. MV conceived of the study, participated in its design and coordination and helped to draft the manuscript. All authors read and approved the final manuscript.
